# Donor-specific assemblies enhance somatic structural variant detection in complex genomic regions

**DOI:** 10.64898/2026.02.20.707061

**Published:** 2026-02-20

**Authors:** Taralynn Mack, Jiadong Lin, Luyao Ren, Min-Hwan Sohn, Anna Minkina, Youngjun Kwon, DongAhn Yoo, Yang Sui, Katherine M Munson, Kendra Hoekzema, F Kumara Mastrorosa, Melanie Sorensen, Marcelo Ayllon, Kaitlyn A Sun, Nidhi Koundiya, Jeffrey Ou, Michelle D Noyes, Adriana Sedeño-Cortés, Amy Leonardson, Caitlin N Jacques, Chris Oliviera, Christian D Frazar, Christina Zakarian, Dana M Jensen, Elliot G Swanson, Erica Ryke, J Thomas Kolar, Jane Ranchalis, Lila Sutherlin, Mitchell R Vollger, Kelsey Loy, Meranda M Pham, Meng-Fan Huang, Natalie YT Au, Patrick M Nielsen, Sean R McGee, Shane Neph, Stephanie Bohaczuk, Tristan Shaffer, Vea Freeman, Yizi Mao, Benjamin Cohen Stillman, Matthew Richardson, Joshua D Smith, Jeffrey M Weiss, Nancy L Parmalee, Chia-Lin Wei, James T Bennett, Andrew B Stergachis, Evan E Eichler

**Affiliations:** 1 Department of Genome Sciences, University of Washington School of Medicine, Seattle, WA, USA; 2 Howard Hughes Medical Institute, University of Washington, Seattle, WA, USA; 3 Division of Medical Genetics, Department of Medicine, University of Washington, Seattle, WA, USA; 4 Center for Developmental Biology and Regenerative Medicine, Seattle Children’s Research Institute, Seattle, WA, USA; 5 The Northwest Genomics Center, University of Washington, Seattle, WA, USA; 6 Department of Human Genetics, University of Utah, Salt Lake City, UT, USA; 7 Brotman Bay Institute for Precision Medicine, Seattle, WA, USA

## Abstract

Structural variants (SVs) contribute substantially to genomic variation and disease, but detecting somatic SVs (sSVs) remains difficult due to reference bias, mosaicism, and enrichment in repetitive regions. Linear reference genomes, like GRCh38 and CHM13, do not fully capture individual genomic structure, which can obscure true somatic variation. Donor-specific assemblies (DSAs) generated from the same genome where sSVs are being assayed provide a personalized alternative, yet their performance for sSV detection has not been systematically assessed. As part of the Somatic Mosaicism across Human Tissues (SMaHT) Network, we benchmark a DSA for sSV discovery in the COLO829 melanoma cell line with a matched normal sample from the same individual. We compare sSV detection across GRCh38, CHM13, and the COLO829BL_DSA using three different sSV callers (Delly, Severus, and Sniffles2) and sequence data from multiple long-read platforms. The COLO829BL_DSA identifies 1.8-fold more manually validated sSVs than linear references, in regions both shared with GRCh38 and CHM13 and unique to the COLO829BL_DSA. Variants detected only with the COLO829BL_DSA are often found in satellite and other repeat-rich regions that are difficult to resolve using standard references. In addition, several COLO829BL_DSA-specific sSVs are located in genes, some of which are associated with cancer. Overall, these results underscore the utility of DSAs in improving sSV detection.

## INTRODUCTION

Structural variants (SVs), defined as genetic alterations of at least 50 base pairs in length, have a well-established role in cancer development (Cosenza et al. 2022; Zhang et al. 2019; Yang and Yang 2023; Lopez et al. 2020). Beyond cancer, SVs have recently been implicated in a broad range of additional diseases, including neurodegenerative disorders (Li et al. 2025; Miller et al. 2021) and age-related clonal hematopoiesis (Loh et al. 2018). However, detecting SVs remains technically challenging. Germline SVs are often large, nonrandomly distributed, and enriched in repetitive and structurally complex genomic regions that are difficult to resolve using short-read sequencing. The landscape of somatic structural variants (sSVs) is less well understood. Detecting sSVs is further complicated by their mosaic nature and potential low variant allele frequency.

Traditional linear human reference genomes (e.g., GRCh38) represent consensus sequences from multiple donors (Lander et al. 2001). They often fail to capture individual-specific SVs, especially within repetitive or highly polymorphic regions—precisely the regions of the genome that are most mutable (Porubsky et al. 2025). This reference bias can obscure true variation and limit the sensitivity of sSV detection. More recently, nearly complete telomere-to-telomere (T2T) human reference genomes (e.g., CHM13) have been produced from a single source material (Nurk et al. 2022). Yet, germline variants present in an individual may still be entirely missed when they occur in genomic regions absent from that reference (Chaisson et al. 2015b).

The development of long-read sequencing technologies has significantly improved the resolution of SVs, enabling more comprehensive detection across all regions of the genome (Chaisson et al. 2015a). In parallel, donor-specific assemblies (DSAs), personalized reference genomes built from an individual donor’s sequencing data, have emerged to enhance SV detection and improve mapping in repetitive genomic regions, allowing both somatic and germline variants to be readily distinguished (Nurk et al. 2022). By more accurately representing the germline genetic background, DSAs can reduce reference bias and improve sensitivity and specificity. Recent work has demonstrated the utility of using a DSA to detect somatic single-nucleotide variation (Sohn et al. 2025), and, more recently, sSVs (Zhang et al. 2025), highlighting the potential of this approach while underscoring the need for broader benchmarking.

Here, as part of the benchmarking efforts of the Somatic Mosaicism across Human Tissues (SMaHT) Network, we assess the utility of using a DSA for detecting sSVs in the well-characterized COLO829 melanoma cell line, which includes matched tumor (COLO829) and normal (COLO829BL) samples (RRID:CVCL_1137). We generated sequencing data from three complementary long-read sequencing platforms: PacBio high-fidelity (HiFi), standard Oxford Nanopore Technologies (STD-ONT), and ultra-long ONT (UL-ONT). These datasets were analyzed with three sSV callers, Delly (Rausch et al. 2012), Severus (Keskus et al. 2024), and Sniffles2 (Smolka et al. 2024), which were chosen due to their performance in previous long-read cancer genome benchmarking studies (Aydin et al. 2025). We use these callers to identify five classes of sSVs: insertions (INS), deletions (DEL), duplications (DUP), inversions (INV), and breakends (BND). Insertions add new genetic sequence, deletions remove sequence, and duplications create additional copies of existing sequence. Inversions flip the orientation of a sequence segment, while breakends represent points of discontinuity in the genome that can indicate translocations or other more complex rearrangements (Feuk et al. 2006). Our findings demonstrate that DSAs are able to capture more true-positive sSVs than traditional references and offer guidance on how to implement DSAs effectively. These insights will inform future work within the SMaHT effort, which will include sequencing of 150 donors across multiple tissues, and will benefit the broader research community as individualized genome assemblies become increasingly accessible and potentially the standard of future clinical care (Miga and Eichler 2023).

## RESULTS

### Construction of a high-quality donor-specific assembly capturing near-T2T completeness

In addition to using the standard genome references for sSV detection, we constructed a DSA from HiFi, UL-ONT, and Hi-C data produced from the normal lymphoblastoid source material (COLO829BL) and the details of this are described elsewhere (Sohn et al. 2025). Briefly, we applied the assembly algorithm Verkko 2.1 to generate a phased diploid genome where two haplotypes were assembled near T2T. The resulting assembly harbored less than 50 gaps per haplotype with an estimated single-nucleotide variant error rate of <1 in a million base pairs, with at least nine non-acrocentric chromosomes represented near T2T in at least one haplotype. We observed that, except for chromosome 21, all acrocentric regions were assembled continuously from the p-arm to the q-arm in both haplotypes with the exception of the rDNA cluster. We also investigated regions of potential errors based on depth of realigned reads and estimated 23.9 Mbp of potential collapse and 6.9 Mbp of misassembly. These numbers are comparable to near-T2T genome assemblies released by international consortia, including the HPRC (Liao et al. 2023) and HGSVC (Logsdon et al. 2025). Comparison to GRCh38 identified 342.8 Mbp of sequence not present in the standard reference genome of which 61% of it was composed of satellite repeats and 22% represented segmental duplications unique to the COLO829BL_DSA. We refer to this alternate reference genome as COLO829BL_DSA to distinguish it from later assemblies of the tumor DNA which is used to validate more complex sSVs.

### sSV detection across references reveals higher sensitivity in COLO829BL_DSA

**Figure 1.**
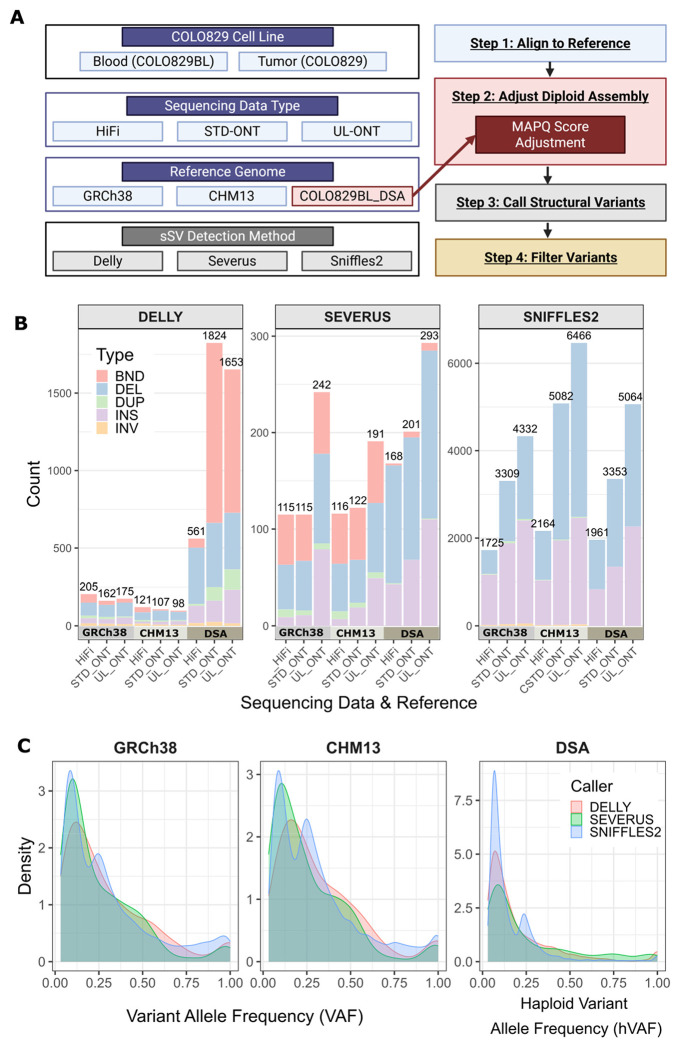
Somatic structural variant (sSV) discovery. **A)** Schematic depicting study design. PacBio high-fidelity (HiFi), standard Oxford Nanopore Technologies (STD-ONT), and ultra-long ONT (UL-ONT) sequencing data for COLO829BL and COLO829 were aligned to three reference genomes: GRCh38, CHM13, and the COLO829BL_DSA. MAPQ values were manually adjusted for the COLO829BL_DSA calls to avoid variant dropout. Three sSV callers, Delly,^15^ Severus,^16^ and Sniffles2,^17^ were used to detect somatic structural variation. **B)** Raw sSV call counts for each caller, reference genome, and type of sequencing data. Color represents the type of sSV. Note sSV counts vary by more than an order of magnitude depending on the caller. **C)** Variant allele frequency (VAF) distributions for GRCh38 and CHM13, and haploid variant allele frequency (hVAF) for COLO829BL_DSA, shown for raw sSVs stratified by reference genome and caller. DSA: donor-specific assembly

Three sSV callers (Delly, Severus, and Sniffles2) were used to call sSVs on the COLO829 data aligned to three references (GRCh38, CHM13, and COLO829BL_DSA) using three sequencing data types PacBio HiFi, STD-ONT, and UL-ONT (Figure 1A). Across all sequencing data types and callers there were 13,975 total sSVs called for GRCh38, 24,396 for CHM13, and 18,061 for COLO829BL_DSA that were 50 bp or longer. Post-calling filtering was performed to remove both sequencing artifacts and germline events. Filtering sSVs in regions of known assembly artifacts or misassembly/collapse removed 7% of sSVs from GRCh38, 31% of CHM13 sSVs, and 14% of COLO829BL_DSA sSVs. Running Phased Assembly Variant (PAV) caller (Ebert et al. 2021) to detect germline SV differences between the COLO829BL_DSA and the respective assemblies resulted in the removal of an additional 16% of GRCh38 variants and 10% of CHM13 sSVs as germline artifacts (Methods, **Supplementary Figure 1A**). After the filtering steps, the remaining calls differed by both caller and reference: Delly (GRCh38: 542, CHM13: 326, COLO829BL_DSA: 4,038), Severus (GRCh38: 472, CHM13: 429, COLO829BL_DSA: 662), and Sniffles2 (GRCh38: 9,366, CHM13: 13,712, COLO829BL_DSA: 10,378) (Figure 1B, **Supplementary Figure 1B**). Delly and Severus detected the most variants using the COLO829BL_DSA, with up to a 15-fold increase in Delly calls. Sniffles2 detected the most variants in CHM13. More variants were detected using ONT across all callers, especially UL-ONT.

sSV types detected also differed by caller, with all five types (INS, DEL, BND, DUP, INV) detected in Delly and Severus and four types (INS, DEL, DUP, INV) detected using Sniffles2 (Figure 1B, **Supplementary Figure 1B**). The COLO829 dataset originates from a single tumor cell, meaning our results contain two classes of sSVs: those that are fixed in the tumor cell line (high variant allele frequency (VAF)) and those that arose during subsequent cell-culture expansion (low VAF). The parameters for all three sSV callers were set to detect both types, ensuring that we capture the full set of differences between COLO829BL and COLO829 (Figure 1C). COLO829BL_DSA calls are reported with haploid variant allele frequency (hVAF) since the sSVs are called from a diploid genome, and COLO829 ploidy has been reported (Sohn et al. 2025).

### Cross-caller and cross-platform sSV integration reveals COLO829BL_DSA-unique events

**Figure 2.**
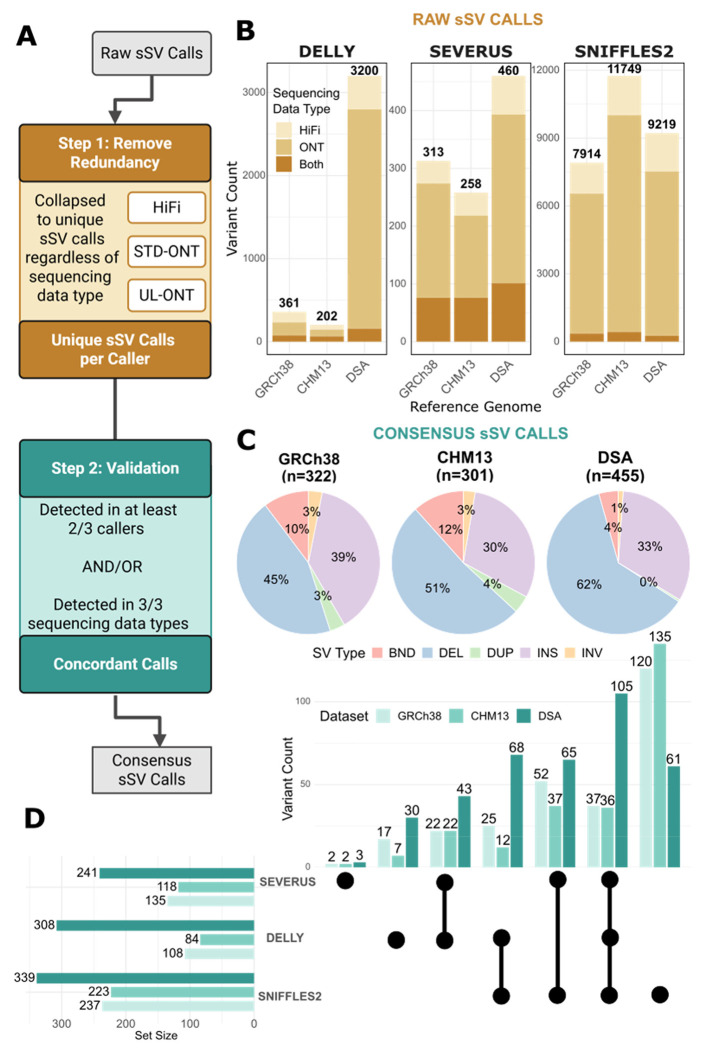
sSV callset integration. **A)** Schematic illustrating the process of integrating raw sSV calls, which involves removing redundancy by sequencing data type and validating each variant through detection by multiple callers or sequencing data types. **B)** Plot summarizing the number of unique variants per caller and reference genome after integrating all sequencing data types (HiFi, STD-ONT, UL-ONT). Bars are colored by the sequencing data types that detected each variant: HiFi, ONT (STD-ONT or UL-ONT), or both. **C)** Pie charts summarizing the number of variants in each reference genome detected by at least two of three sSV callers or by all three sequencing data types. Slice color indicates structural variant type: pink - BND (breakend), blue - DEL (deletion), green - DUP (duplication), purple - INS (insertion), and orange - INV (inversion). **D)** Upset plot indicating which callers detected each variant in the consensus callset and the total number of variants contributed by each sSV caller. Bar color corresponds to the reference genome.

To produce a consensus sSV callset, we applied a two-stage integration strategy comprising (i) redundancy reduction and (ii) cross-caller and cross-sequencing-platform validation (Figure 2A). Using Truvari (English et al. 2022), the filtered variants were collapsed into one callset per reference genome regardless of sequencing data type (Methods). Most variants were detected by only one platform, with 95% of calls unique to either HiFi or ONT (STD-ONT and/or UL-ONT) across all references, with ONT-only accounting for most variants (79%). ONT-only variants comprised 36% for Delly, 63% for Severus, and 78% for Sniffles2, whereas HiFi-only variants represented a smaller fraction (36%, 13%, and 17%, respectively) (Figure 2B). After removing redundancies, the total remaining calls for each caller were as follows: Delly - GRCh38: 361, CHM13: 202, COLO829BL_DSA: 3200; Severus - GRCh38: 313, CHM13: 258, COLO829BL_DSA: 460; and Sniffles2 - GRCh38: 7,914, CHM13: 11,749, COLO829BL_DSA: 9,219 (Figure 2B). The COLO829BL_DSA continued to show the greatest sSV count for Delly and Severus, while CHM13 had the greatest sSV count for Sniffles2 (Figure 2B).

After reduction, calls were integrated into one callset per reference genome. An sSV was included in the consensus dataset if it was detected by at least two of the three sSV callers, or if it was detected across all three sequencing data types for a single caller. This integration yielded 322 variants for GRCh38, 301 variants for CHM13, and 455 variants for COLO829BL_DSA. Insertions, deletions, breakends, duplications, and inversions were observed across all three references (Figure 2C). The largest category of variants in the consensus dataset (34%) was supported by only Sniffles2, which were later discovered to be all false positives, while the smallest category (<1%) consisted of variants detected exclusively by Severus. Sniffles2 contributed the largest number of variants to the consensus callset, followed by Delly and then Severus (Figure 2D). When considering COLO829BL_DSA calls alone, the largest fraction of variants was supported by all three sSV callers, as a smaller proportion of them are supported by Sniffles2 alone (Figure 2D).

### *Manual validation confirms that* COLO829BL_DSA *improves detection of true sSVs*

**Figure 3.**
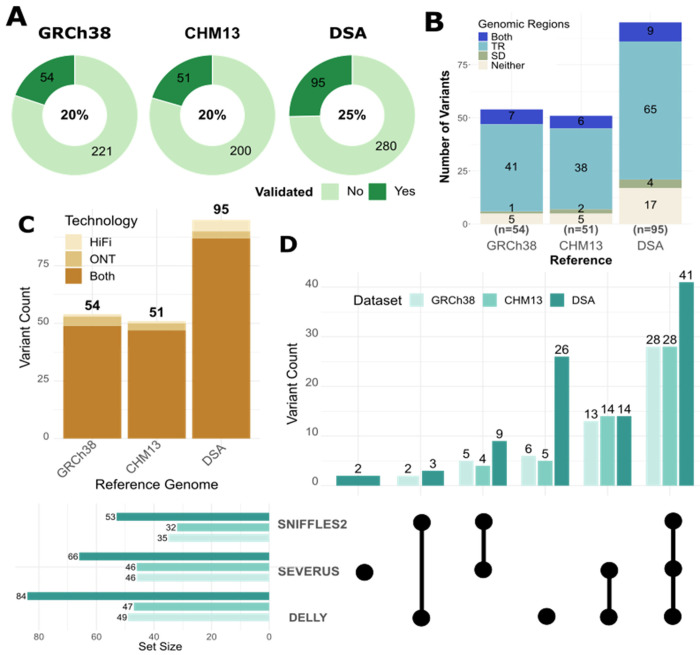
Reference comparisons. **A)** Percentage of variants from the consensus set manually validated as true positive calls across the different reference genomes. **B)** Plot showing the number of sSVs mapping to tandem repeat (TR) or segmental duplication (SD) regions of the genome across the three different reference genomes. **C)** Sequencing data types that called each true positive variant across HiFi and ONT. **D)** Combinations of sSV callers that called each true positive variant.

Following the curation of the consensus dataset, we identified a subset (excluding BNDs) that we were able to confidently manually validate by visual inspection of the underlying genome alignment and sequencing reads. There were 54 true positive variants for GRCh38, 51 for CHM13, and 95 for the COLO829BL_DSA which corresponded to 20%, 20%, and 25% of the candidate variants, respectively (Figure 3A). The majority of manually validated variants mapped to tandem repeat regions across all assemblies, accounting for 89% in GRCh38, 86% in CHM13, and 65% in the COLO829BL_DSA (Figure 3B).

Most true positive variants (87%) were called by both HiFi and ONT platforms (Figure 3C). The largest category of the manually validated variants (49%) was confirmed by all three sSV callers, while no manually validated variants were detected by Sniffles2 alone (Figure 3D). Severus contributed the largest percentage of true positive calls to the dataset (26%), followed by Severus (34%), and then Delly (39%) (Figure 3D).

To provide further validation, we also constructed a tumor-specific assembly (COLO829_DSA) with the existing long reads and compared it to the COLO829BL_DSA (Methods). The reads used had a read N50 of 58.4 kbp, with 18.9× coverage from reads longer than 100 kbp and a total coverage of 66.6×. Although highly fragmented and incomplete, the resulting cancer cell line genome assembly had a contig N50 of 134.5 Mbp, with a QV of 57.9 based on HiFi reads. We compared COLO829_DSA versus COLO829BL_DSA and called sSVs using PAV (Ebert et al. 2021). Of the manually validated variants, 42% were supported by this assembly-to-assembly comparison, further evidence of the sSVs detected by the read-based callers. Notably, the average hVAF detected by both the read-based and assembly-based approaches was 63%, compared to 35% for variants not detected by the assembly-based method, indicating that PAV is more likely to capture sSVs present at higher VAFs. This is expected as PAV was designed to call germline variants and not somatic mutations; its use here, however, confirms high-frequency events at the assembly level.

### COLO829BL_DSA uncovers unique sSVs missed by standard references

**Figure 4.**
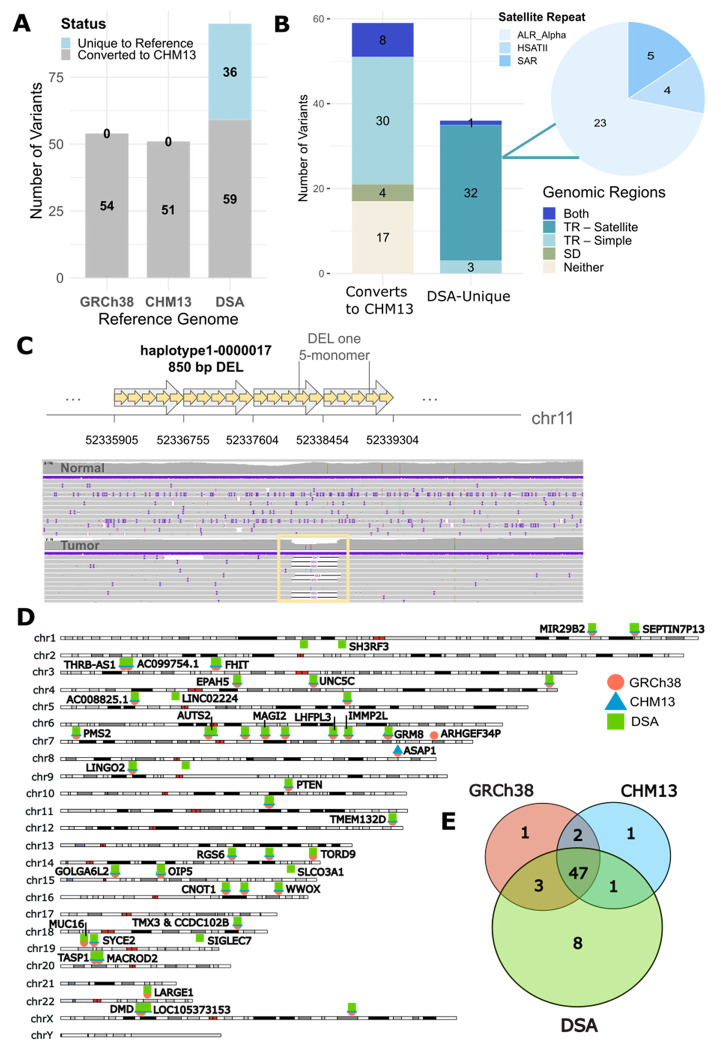
Sequence properties and genome-wide distribution of sSVs. **A)** Plot showing the number of sSV calls for each reference genome converted to CHM13 coordinates (displayed in Figure 3C,D) compared to the percentage that are unique to that reference genome (not displayed in Figure 3C,D). **B)** Genomic regions of COLO829BL_DSA variants for either COLO829BL_DSA variants converted to CHM13 or those unique to the COLO829BL_DSA. For those unique to the COLO829BL_DSA, a pie chart of the satellite subtypes is shown, with the vast majority as alpha satellites. **C)** Example of a deletion in a COLO829BL_DSA-unique alpha satellite region. **D)** Ideogram showing the location of sSV calls across the genome for all manually validated sSVs able to be mapped to CHM13 coordinates. Each reference is represented by a different color and shape, and overlap occurs when the shapes intersect. For any variants that overlap genes, the gene name is notated. **E)** Venn diagram displaying the number of overlapping variants between the different reference genomes when the sSV calls are mapped to CHM13 coordinates.

To compare sSV calls across the unique coordinate systems of the references, we implemented a graph-based approach (Methods) to remap variants. When all 95 manually validated sSVs from COLO829BL_DSA were converted to CHM13, 36 variants failed to convert using this approach. Further investigation showed that 36/36 (100%) of these variants are unique to the COLO829BL_DSA and would not be detected by the other reference genomes (Figure 4A). As expected, the majority of COLO829BL_DSA-unique variants mapped to repetitive regions, with 86% located in satellite repeats, and of those satellites, 75% corresponded to alpha satellites, including sSVs mapping to higher-order alpha satellite DNA (Figure 4B–C). sSVs were distributed across the chromosomes, with the highest number of sSVs detected on chromosomes 7 and X (Figure 4D). Of the 63 unique events mapping to CHM13 coordinates, 13% were detected using only COLO829BL_DSA, while 75% were detected by all three references. Four variants were not detected by the COLO829BL_DSA and were detected by GRCh38 and/or CHM13 (Figure 4E). For the 63 total sSVs converted to CHM13, there was no significant difference in length or VAF between those sSVs detected by only the COLO829BL_DSA compared to those shared across references.

To identify variants of potential functional consequence, we focused on sSVs mapping near or potentially disrupting protein-coding genes. Among the sSVs detected by all three references, 39 out of 47 are located within genes (Table 1, Methods), and of those, six impact the coding regions of the genes, *LHFPL3, PTEN, GOLGA6L2, CNOT1, TMX3-CCDC102B*, and *DMD*. Four of those, *LHFPL3* (Zhang et al. 2020), *PTEN* (Yehia et al. 2025; Khasarah et al. 2025), *GOLGA6L2* (Sarker et al. 2025), and *TMX3-CCDC102B* (Luo et al. 2023; Si et al. 2022) have known links to cancer. Additional sSVs identified impact regulatory regions (Table 1).

**Figure 5.**
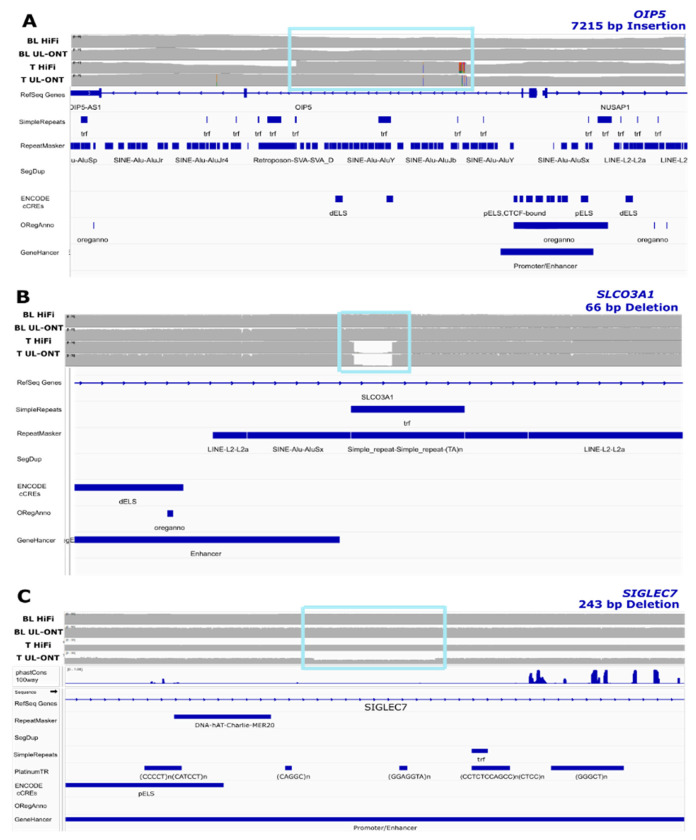
COLO829BL_DSA-only sSVs near regulatory regions. Example of COLO829BL_DSA-only manually validated deletion on one haplotype impacting **A)** *OIP5*, **B)** *SLCO3A1*, and **C)** *SIGLEC7*. BL: blood; T: tumor

Of those sSVs detected only by the COLO829BL_DSA, 5/8 overlap genes and are intronic. Three of these overlap potential regulatory regions, *OIP5, SLCO3A1*, and *SIGLEC7*; *SIGLEC7* (Benthami et al. 2025; Thangaraj et al. 2025) has known links to cancer (Figure 5, Table 2).

## DISCUSSION

Here, we evaluated the utility of DSAs for detecting sSVs in the well-characterized COLO829 melanoma cell line, comparing results across standard reference genomes (GRCh38, CHM13) and both HiFi and ONT long-read sequencing platforms. We demonstrate that using the COLO829BL_DSA captures approximately 1.8-fold more manually validated sSVs than either GRCh38 or CHM13. Importantly, this improvement reflects increased sensitivity both within genomic regions shared across references and within the COLO829BL_DSA-specific sequence that is absent from standard reference assemblies. Nearly all COLO829BL_DSA-unique sSVs (99%) localize to a tandem repeat or segmental duplication region of the genome, which are notoriously difficult to capture with traditional references. Beyond the uniquely represented sequence, the COLO829BL_DSA also improved sSV detection within regions shared across the references, identifying eight additional validated variants that were missed when using GRCh38 or CHM13. Together, these results highlight the value of individualized genome assemblies for more completely capturing the full landscape of somatic variation, particularly in complex and repetitive genomic regions.

We also provide practical suggestions for how to handle the complexities of working with DSAs. Because DSAs are diploid, reads originating from regions that are nearly identical between haplotypes can map ambiguously to both copies, leading to reduced mapping quality scores and dropout of true variants. We demonstrate that manually adjusting mapping quality scores for reads aligned to the COLO829BL_DSA effectively mitigates this issue. This approach was previously demonstrated on somatic single-nucleotide variants (Sohn et al. 2025), but we extend its applicability to sSVs. Recent work has addressed this challenge by analyzing each haplotype independently and merging calls downstream (Zhang et al. 2025), an effective strategy; however, our approach offers a substantially simpler workflow and allows sSVs to be called directly from the phased diploid assembly.

Furthermore, we demonstrate the utility of pangenome graph-based methods to map sSVs from the COLO829BL_DSA to common coordinate systems, such as CHM13. This approach enabled comparison of sSVs across references and allowed us to quantify the degree of overlap and uniqueness for each reference genome. The high proportion of COLO829BL_DSA variants that could not be successfully lifted over, especially in satellite and repeat-rich regions, reinforces the idea that linear references often miss biologically relevant variation.

Our work also shows the importance of manual validation in high-complexity regions. While 71% of the total consensus sSVs across reference genomes turned out to be false positives, we identified a subset of true-positive sSVs supported by individual sequencing read alignments. To improve efficiency in future studies, we propose increasing the clustering threshold for variant proximity in high-complexity regions (e.g., >500 bp) during filtering, which may help consolidate nearby calls and reduce the burden of manual review.

Our analysis also highlights the need for using multiple sSV callers. No single tool was able to comprehensively detect all high-confidence sSVs; instead, cross-caller validation provided stronger support and helped prioritize true somatic events. This is especially true for sSVs, which frequently occur at low variant allele fractions, complicating their discrimination from sequencing noise. Through the manual validation of the consensus callset, we observed substantial differences in caller-specific reliability for calls across all sequencing data types but only one sSV caller.

No variants detected exclusively by Sniffles2 could be manually validated, while 29% of those called by Severus exclusively and 69% of those called exclusively by Delly could. This disparity highlights marked differences in precision across sSV callers and suggests that Delly-only calls are substantially more reliable within this dataset. Our results also demonstrate that Sniffles2 only calls could be eliminated from the consensus dataset to reduce the burden of manual review without losing any substantial signal.

We further observed that >95% of variants in the consensus dataset detected exclusively in either HiFi or ONT sequencing data, but not supported across both technologies, failed manual validation, indicating that cross-platform support is a strong indicator of call reliability. This suggests that requiring concordance between HiFi and ONT data could serve as an effective filtering criterion to reduce false positives in sSV callsets. However, each long-read technology has distinct strengths and limitations (Wang et al. 2025; Harvey et al. 2023), and strict enforcement of cross-platform support would come at the cost of sensitivity. Notably, applying such a criterion would have resulted in the missed cancer-associated gene fusion between *TMX3* and *CCDC102B* since this was only detected in UL-ONT data. This underscores the importance of balancing specificity and sensitivity and highlights the continued need for orthogonal validation and manual review, particularly for variants of potential functional relevance.

Our analysis identified 17 sSVs that map to known cancer-associated genes; 15 of these were detected by all three references, and two were only identified by the COLO829BL_DSA: *OIP5* and *SIGLEC7*. *OIP5*, which is located at the centromere, resides in a genomic region that is particularly prone to being overlooked by sSV calling approaches based on traditional references. Functionally, OIP5 plays a key role in mitosis and is frequently overexpressed in tumor tissues (Pan et al. 2023). *SIGLEC7* encodes an inhibitory immune checkpoint receptor and can promote immune evasion in cancer (Benthami et al. 2025; Thangaraj et al. 2025). Notably, *SIGLEC7*-associated sSVs have not been reported in prior COLO829 analyses. Collectively, these findings demonstrate that DSA-based references not only increase the overall number of detectable sSVs within DSA-unique genomic regions but also enhance detection in less complex regions and uncover previously unreported alterations in established cancer-associated genes.

A limitation of this analysis is its emphasis on specificity over sensitivity. Previous work (Espejo Valle-Inclan et al. 2022), reported 68 experimentally validated sSVs in COLO829. Our analysis recovered 85% of these variants when restricted to non-BND variants larger than 50 bp. The previous study (Espejo Valle-Inclan et al. 2022) employed a more comprehensive sequencing strategy and included PCR-based validation, whereas our experimental design was more limited in scope. Despite these limitations, using the COLO829BL_DSA resulted in the detection of 95 sSVs, compared with only 51-54 when using GRCh38 or CHM13, demonstrating improved performance even under a less exhaustive validation framework. Overall, while our approach may miss some sSVs, the variants it identifies are highly reliable. However, establishing this confidence relied on manual validation, which is not scalable and may introduce subjective bias.

In summary, our study demonstrates that DSAs substantially improve the detection of sSVs, capturing both variants in shared genomic regions and those located in repetitive or DSA-unique sequences that are missed by standard references. Beyond increasing the total number of detectable sSVs, DSAs reveal alterations in cancer-associated genes, which may have functional consequences. We also provide practical suggestions to address DSA challenges and improve sSV calling. These findings underscore the value of phased, haplotype-resolved assemblies for comprehensive sSV discovery and suggest that broader adoption of DSAs could enhance the detection of functionally relevant structural variation in cancer and other complex genomic contexts. Moving forward, efforts within the SMaHT Network will extend these analyses across multiple tissues and donors, providing a more complete understanding of the normal sSV landscape in humans.

## METHODS

### Sequencing Data Generation

Sequencing data for both COLO829 and its matched normal, COLO829BL, were sourced from the SMaHT benchmarking effort (The SMaHT Network and Abyzov 2025). Sequencing data were generated using PacBio HiFi, STD-ONT, and UL-ONT platforms. Comprehensive details for COLO829BL/COLO829 cell culture, library preparation, and sequencing are described in the SMaHT flagship paper (Coorens et al. 2025). The corresponding data can be accessed at https://data.smaht.org.

### Constructing the COLO829BL and COLO829 DSAs

To construct the DSA for COLO829BL, we used 60× coverage of PacBio HiFi and ONT data, as well as 30× Hi-C coverage. ONT reads were downsampled to enrich for the longest reads, and phased assembly was performed using Verkko (version 2.1) (Rautiainen et al. 2023). Post-assembly, we applied the same contamination filtering pipeline involving NCBI FCS (version 0.4.0 beta) (Astashyn et al. 2024), adapter screening, and BLAST-based identification and removal of mitochondrial, Epstein–Barr virus, and rDNA sequences. This process produced decontaminated, haplotype-resolved assemblies. Detailed methods for DSA construction and quality evaluation are described here (Sohn et al. 2025).

For the tumor assembly (COLO829), all available ONT sequencing data, including R9 and R10 chemistries as well as STD-ONT and UL-ONT reads, were used. The initial dataset had a read N50 of 60.041 kbp, with 24.788× coverage from reads longer than 100 kbp and a total coverage of 88.827×. To improve base-level accuracy, reads were processed using fastplong (version 0.2.2) (Chen 2023), applying both 5′ and 3′ trimming based on a mean read base quality threshold of Q20. After trimming, the dataset had a read N50 of 58.440 kbp, with 18.951× coverage from reads longer than 100 kbp and a total coverage of 66.632×.

A pseudo-phased assembly was generated using hifiasm (version 0.25.0) (--dual-scaf --telo-m CCCTAA) (Cheng et al. 2021). To remove potential contaminant sequences, including nonhuman sequences, Epstein–Barr virus, and residual adapter sequences, the assemblies were screened using NCBI FCS (version 0.4.0 beta) (Astashyn et al. 2024) in combination with BLAST(version 2.15) (Camacho et al. 2009).

### Alignment

All three types of raw sequencing reads (HiFi, STD-ONT, and UL-ONT), for both COLO829BL and COLO829, were aligned to each reference genome (GRCh38, CHM13, and COLO829BL_DSA) using pbmm2 (version 1.13.1) for HiFi reads (pbmm2 align –preset CCS) and minimap2 (version 2.28) for STD-ONT and UL-ONT reads (minimap2 -ax map-ont).

### Mapping Quality Score Adjustment

Aligning the sequencing reads onto the diploid COLO829BL_DSA would result in a mapping quality (MAPQ) score of zero, since the read can map to two different locations, representing each haplotype. This can result in variants being discarded in downstream sSV calling due to MAPQ filters by variant callers. To avoid this issue, we manually changed the MAPQ values for the COLO829BL_DSA reads to 60, as further described here (Sohn et al. 2025).

### sSV Calling

For each combination of COLO829BL or COLO829 data, type of sequencing data (HiFi, STD-ONT, and UL-ONT), and reference genome (GRCh38, CHM13, and COLO829BL_DSA), three different sSV callers were used to call sSVs. Delly (version 1.5.0) (Rausch et al. 2012) somatic mode was used with the blood and tumor datasets run together and maximum VAF threshold set at 1. Severus (version 1.5) (Keskus et al. 2024) was used in tumor-normal mode with the blood and tumor datasets with the maximum VAF threshold set at 1. For Sniffles2 (version 2.7.1) (Smolka et al. 2024), COLO829BL and COLO829 data were run separately using both germline and mosaic mode and the resulting callsets were combined. Any variants detected in COLO829BL were removed from the COLO829 dataset using Truvari bench (70% sequence similarity and 500 bp distance) (English et al. 2022).

### sSV Filtering

To remove artifacts from our raw sSV calls, we excluded variants called in regions of known artifacts, misassembly, or collapse for all three reference genomes. We exclude GRCh38 sSVs in gap or telomere regions (within 10 kbp to telomere) from UCSC Genome Browser, accounting for 218 Mbp. CHM13 sSVs in acrocentric short arms, centromeres, and satellite regions were excluded except for monomeric alpha-Sat (https://s3-us-west-2.amazonaws.com/human-pangenomics/T2T/CHM13/assemblies/annotation/chm13v2.0_censat_v2.1.bed) (Logsdon et al. 2025; Sui et al. 2026). We created a nonredundant BED file with BEDTools, resulting in 246 Mbp of masked reference loci. These regions showed an excessive number of variants, specifically in human satellite repeat (e.g., HSat3, etc.), that are likely to be mapping artifacts. For COLO829BL_DSA, we removed any SVs called in regions of known misassembly or collapse. These regions were determined by NucFreq (https://github.com/mrvollger/NucFreq). For NucFreq, the HiFi reads were aligned to the phased diploid COLO829BL_DSA genome containing all haplotypes with minimap2. Then, to remove inherent germline differences between COLO829BL and GRCh38/CHM13, we ran PAV (Ebert et al. 2021) between the assemblies and removed any calls that overlapped from our downstream analyses.

### sSV Callset Integration

After the raw sSV callsets were created, variants were collapsed into one dataset per genome reference and caller regardless of sequencing data type. This process was done using Truvari collapse (70% sequence similarity and 500 bp distance) (English et al. 2022) on a merged VCF of the calls for HiFi, STD-ONT, and UL-ONT with sSV type ignored. Each of these collapsed datasets was then collapsed a second time to create one callset per reference genome using the same parameters, regardless of sSV caller. sSVs present in at least two out of three sSV caller datasets or three out of three sequencing data types for a single caller were considered high-confidence and are present in the consensus dataset.

### Manual Validation

SVs in the consensus set were manually evaluated through visual inspection of sequencing data and genome assemblies. Nucleotide frequency (NucFreq) (Vollger et al. 2019) plots were examined to assess local assembly quality and to identify regions with atypical base composition patterns that could indicate assembly errors. sSVs were further validated by visualizing aligned sequencing reads in the Integrative Genomics Viewer (IGV) (Robinson et al. 2011). sSVs were considered supported when read-level evidence in IGV was concordant with the predicted sSV and no obvious assembly artifacts were indicated by NucFreq analysis.

### Coordinate Conversion Between Assemblies

To compare the sSV calls between reference genomes, coordinates needed to be converted to a common coordinate system. Here we used CHM13. To convert the COLO829BL_DSA coordinates, we used a donor-specific pangenome graph constructed with Minigraph-Cactus (Hickey et al. 2024). It contains GRCh38, CHM13, and the two haplotypes of COLO829BL, allowing for bidirectional coordinate translation between assemblies and identification of COLO829BL_DSA-specific intervals. This process is described in more detail here (Sohn et al. 2025).

### Identifying Tandem Repeat and Segmental Duplication Regions

Repeat element annotations were added using Rhondite (10.5281/zenodo.6036498), which combines RepeatMasker (version 4.1.5) (https://github.com/Dfam-consortium/RepeatMasker), Tandem Repeats Finder (TRF) (version 4.1.0) (Benson 1999), and DupMasker in a snakemake workflow. Detailed methods are available here (Sohn et al. 2025).

### Segmental Duplication Annotation

Repetitive sequences in each genome assembly were identified and masked using three complementary tools. RepeatMasker (version 4.1.5) (https://github.com/Dfam-consortium/RepeatMasker) was run with the parameters “*-s -e ncbi -xsmall -species human [asm.fa]*” followed by TRF (version 4.1.0) (“*trf [asm.fa] 2 7 7 80 10 50 2000 -l 30 -h -ngs*”) (Benson 1999) and WindowMasker (“*windowmasker -mk_counts -mem 16384 -smem 2048 -infmt fasta -sformat obinary -in [asm.fa] -out [asm.count]* && windowmasker -infmt fasta -ustat [asm.count] -dust T -outfmt interval -in [asm.fa] -out [asm.interval]”) (Morgulis et al. 2006) to comprehensively mask the repeats. The repeat annotations from all three tools were integrated, and the merged BED intervals were used to softmask the assemblies. Segmental duplications were then identified on these masked assemblies using SEDEF (version 1.1) (Numanagić et al. 2018). For subsequent analyses, only duplications exceeding 1 kbp in length, with >90% sequence identity and <70% satellite DNA content, were retained.

### Identifying Gene Overlap and Annotations

To determine whether the manually validated sSVs overlapped annotated genes, sSV coordinates were compared against gene annotations using the UCSC Genome Browser (https://genome.ucsc.edu/). sSV intervals were visualized and queried within CHM13 and/or GRCh38, and overlap with known genes was assessed based on UCSC gene annotation tracks. An sSV was considered gene-overlapping if any portion of the variant interval intersected the genomic coordinates of an annotated gene, including exonic or intronic regions. To identify SV location in relation to exons/introns, promoters/enhancers, and regulatory elements, we integrated published datasets from UCSC Genome Browser tracks, including candidate cis-regulatory elements (ENCODE Regulation, ENCODE cCREs, ORegAnno, GeneHancer) and ENCODE histone marks (H3K27Ac, H3K4Me1, H3K4Me3).

## Figures and Tables

**Table 1.** sSVs (47) detected by all three reference genomes in CHM13 coordinates.

Chr	Start	End	Length (bp)	Type	Avg CHM13 VAF	Gene	Location (ENCODE)
1	207054643	207088259	33616	DEL	0.25	*MIR29B2CHG*	Intronic
1	223647744	223801247	153503	DUP	0.32	*CNIH3*	Intronic
1	223783915	223787137	3222	DEL	0.35	*CNIH3*	Intronic
3	24528015	24529090	1075	INV	0.46	*THRB*	Intronic
3	26625031	26625606	575	INV	0.42	*AC099754.1*	Intronic
3	60154812	60243152	88340	DEL	0.18	*FHIT*	Intronic
3	60188785	60260598	71813	DEL	0.30	*FHIT*	Intronic
3	60929642	61070679	141037	DEL	0.27	*FHIT*	Intronic
4	68783148	68783149	84	INS	0.47	*EPHA5*	Intronic
4	98503674	98505871	2197	DEL	0.16	*UNC5C*	Intronic, Enhancer, Regulatory Regions
4	190421537	190421538	61	INS	0.52	None	Intergenic
5	28893439	29068757	175318	DEL	0.53	*LINC02109*	Intronic
5	111723427	111733271	9844	DEL	0.28	None	Intergenic, Enhancer, Regulatory Regions
7	6090243	6090244	71	INS	0.42	*PMS2*	3’UTR
7	57647407	57679944	32537	DEL	0.47	None	Intergenic
7	71862133	71990480	128347	DEL	0.16	*AUTS2*	Intronic, Regulatory Regions
7	79604442	79704838	100396	DEL	0.17	*MAGI2*	Intronic, Regulatory Regions
7	79812784	79880280	67496	DEL	0.26	*MAGI2*	Intronic, Regulatory Regions
7	87465292	87474334	9042	DUP	0.13	None	Intergenic, Regulatory Regions
7	106159459	106286673	127214	DUP	0.39	*LHFPL3*	Exonic, Regulatory Regions
7	112071599	112095496	23897	DEL	0.12	*IMMP2L*	Intronic, Regulatory Regions
7	112147097	112148225	1128	DEL	0.29	*IMMP2L*	Intronic
7	127770165	127839120	68955	INV	0.41	*GRM8*	Regulatory Regions
9	28017717	28141137	123420	DEL	0.45	*JAZF1*	Intronic, Regulatory Regions
9	28042441	28069808	27367	INV	0.48	*JAZF1*	Intronic, Regulatory Regions
10	88824448	88836465	12017	DEL	0.86	*PTEN*	Exonic, Enhancer, Regulatory Regions
11	81009351	81317624	308273	DEL	0.38	None	Intergenic
11	81541347	81573404	32057	DEL	0.20	None	Intergenic, Regulatory Regions
12	129318482	129318483	356	INS	0.64	*TMEM132D*	Intronic
14	66753567	66753568	98	INS	0.99	*RGS6*	Intronic
14	81341023	81341105	82	DEL	0.17	None	Intergenic
15	21173737	21178284	4547	DEL	0.17	*GOLGA6L2*	Exonic, Regulatory Regions
15	21320936	21482011	161075	INV	0.40	*MKRN3*	Intronic
16	64386222	64425003	38781	DEL	1	*CNOT1*	Exonic, Regulatory Regions
16	71620937	71623422	2485	DEL	0.17	None	Intergenic
16	84950985	85117389	166404	DEL	0.54	*WWOX*	Intronic, Regulatory Regions
18	68928344	68931708	3364	DEL	0.10	*TMX3* & *CCDC102B*	Exonic, Promoter/Enhancer, Regulatory Regions
19	13042080	13042389	309	DEL	0.07	*SYCE2*	Intronic
20	13223439	13226811	3372	DEL	0.53	*TASP1*	Intronic, Regulatory Regions
20	15032189	15083181	50992	DEL	0.24	*MACROD2*	Intronic, Regulatory Regions
20	15069862	15083117	13255	DEL	0.41	*MACROD2*	Intronic, Regulatory Regions
X	30776959	30796209	19250	DEL	0.56	*DMD*	Exonic, Enhancer, Regulatory Regions
X	30881230	31618768	737538	DEL	0.56	*DMD*	Intronic
X	31657970	31873134	215164	DEL	0.52	*DMD*	Intronic
X	31678648	31781312	102664	DEL	0.92	*DMD*	Intronic, Regulatory Regions
X	33639784	33642710	2926	DEL	0.98	*LOC105373153*	Intronic
X	113470897	113470963	66	DEL	0.14	None	Intergenic, Regulatory Regions

**Table 2.** sSVs (8) in locations shared by all references detected only by COLO829BL_DSA.

Chr	Start	End	Length (bp)	Type	Avg DSA hVAF	Gene	Location (ENCODE)
1	223203354	223203355	85	INS	0.94	*SEPTIN7P13*	Intronic, Regulatory Regions
2	94781130	94788844	7714	DEL	0.30	None	Intergenic
2	109659823	109659824	330	INS	0.74	*SH3RF3*	Intronic
5	44794227	44794228	6050	INS	0.19	None	Intergenic
9	48741978	48742317	339	DEL	0.35	None	HSat3
15	39128147	39128148	7215	INS	0.39	*OIP5*	Intronic, Regulatory Regions
15	89718730	89718796	66	DEL	0.87	*SLCO3A1*	Intronic, Regulatory Regions
19	54231929	54232172	243	DEL	0.19	*SIGLEC7*	Intronic, Promoter/Enhancer, Regulatory Regions

## Data Availability

The SMaHT COLO829 benchmarking data can be accessed from the SMaHT Data Portal at https://data.smaht.org/. The data is accessible to anyone after the creation of an account.
